# Preclinical evaluation of [^58m^Co]Co-DOTA-PSMA-617 for Auger electron therapy of prostate cancer

**DOI:** 10.1038/s41598-023-43429-8

**Published:** 2023-11-01

**Authors:** Christina Baun, Johan Hygum Dam, Malene Grubbe Hildebrandt, Jesper Dupont Ewald, Bjarne Winther Kristensen, Vigga Sand Gammelsrød, Birgitte Brinkmann Olsen, Helge Thisgaard

**Affiliations:** 1 Department of Nuclear Medicine, Odense University Hospital, Kløvervænget 47, 5000 Odense C, Denmark; 2 Department of Clinical Research, University of Southern Denmark, Odense, Denmark; 3 Center for Personalized Response Monitoring in Oncology (PREMIO), Odense University Hospital, Odense, Denmark; 4 Centre for Innovative Medical Technology, Odense University Hospital, Odense, Denmark; 5 Department of Pathology, Odense University Hospital, Odense, Denmark; 6 Department of Surgical Pathology, Zealand University Hospital, Roskilde, Denmark

**Keywords:** Cancer, Diseases, Medical research, Molecular medicine, Oncology

## Abstract

Prostate-specific membrane antigen (PSMA), highly expressed in prostate cancer, is a promising target for radionuclide therapy. Auger electron-emitting radionuclides are well suited for targeted radionuclide therapy if they can be delivered close to the DNA of the targeted cells. This preclinical study evaluated the theranostic pair [^55/58m^Co]Co-DOTA-PSMA-617 for PET imaging and Auger electron therapy of prostate cancer. [^58m^Co]Co-DOTA-PSMA-617 was successfully prepared with > 99% radiochemical yield and purity. In vitro, uptake and subcellular distribution assays in PSMA-positive prostate cancer cells showed PSMA-specific uptake with high cell-associated activity in the nucleus. Incubation with [^58m^Co]Co-DOTA-PSMA-617 reduced cell viability and clonogenic survival in a significant dose-dependent manner (p < 0.05). Biodistribution of xenografted mice showed high specific tumor uptake of the cobalt-labeled PSMA ligand for all time points with rapid clearance from normal tissues, which PET imaging confirmed. In vivo*,* therapy with [^58m^Co]Co-DOTA-PSMA-617 in tumor-bearing mice demonstrated significantly increased median survival for treated mice compared to control animals (p = 0.0014). In conclusion, [^55/58m^Co]Co-DOTA-PSMA-617 displayed excellent in vitro and in vivo properties, offering significant survival benefits in mice with no observed toxicities.

## Introduction

The prostate-specific membrane antigen (PSMA) is a type II membrane glycoprotein overexpressed in prostate cancer and further upregulated in metastatic disease^1^. Patients with advanced metastatic castration-resistant prostate cancer have a poor prognosis despite the development in treatment options^2^. Hence, PSMA has gained considerable interest as a potential target for radionuclide therapy in this patient group^3^. Clinical trials with [^177^Lu]Lu-DOTA-PSMA-617 or PSMA-I&T as beta-emitting radionuclide therapy for metastatic castration-resistant prostate cancer has shown promising potential but also a high risk of side effects^4,5^. In addition, alpha-emitting therapy with radium-223 dichloride (Xofigo) is in clinical use for the treatment of bone metastases in prostate cancer patients, and other alpha-emitting therapies are currently under investigation^3,6,7^. However, despite the development of targeted radionuclide therapy, none of the existing treatments seem optimal due to heterogeneity in the therapeutic effect and adverse events, besides logistic and regulatory challenges^4,6,7^. Hence, there is an urgent need to develop or refine new targeted treatments with minimal side effects.

Auger electron-emitting radionuclides have shown new opportunities for targeted radionuclide therapy if they can be delivered close to the DNA of the targeted cells^8^. Auger electrons (AEs) are emitted in a cascade and have energy deposited over a short range (nm to µm), thus resulting in high linear energy transfer (4–26 keV/µm) around the decay site^8,9^. Several investigations of targeted radionuclide therapy with AE-emitting radionuclides have demonstrated promising therapeutic effects and low toxicity compared to beta- and alpha-therapy^8–11^.

PSMA-617 is internalized in prostate cancer cells by endocytosis and, thus, an interesting carrier for AE-emitting radionuclides^3^. However, only a few studies have investigated AE therapy with PSMA and only in preclinical settings, but with promising effects and low toxicity^8,12,13^.

The theranostic pair, cobalt-55 (t_1/2_ = 17.53 h, β +  = 77%, Eγ = 931.1 keV, Iγ = 75%) for PET-imaging and cobalt-58m (t_1/2_ = 9.04 h, IT = 100%) for AE therapy, have gained increasing interest and found promising due to their identical chemical properties and high in vivo radionuclide-chelator complex stability^11,14,15^. The AE-emitting radionuclide cobalt-58m can be produced with a low-energy cyclotron in high yields^14^. AE therapy with cobalt-58m has shown a significant therapeutic effect in vitro and promising dosimetry^11,16^, but no in vivo therapy has been published to our knowledge. For PET imaging, cobalt-55 has already demonstrated excellent imaging properties compared to other PET-imaging agents^17–22^.

Here, we evaluated the theranostic pair [^55/58m^Co]Co-DOTA-PSMA-617 for PET-imaging and AE therapy for prostate cancer in vitro and in vivo.

## Results

### Radiolabeling and in vitro evaluation

Radiolabeling of [^58m^Co]Co-DOTA-PSMA-617 (1555 ± 176 MBq) was performed in high radiochemical yield and purities above 99% (n = 4) and apparent molar radioactivity of 84.0 ± 6.8 MBq/nmol at the end of synthesis (EOS). In addition, the radio complex of [^58m^Co]Co-DOTA-PSMA-617 released approx. 2.4% radiocobalt (cobalt-58m & cobalt-58g) within the first 24 h with no further release up to 8 days as evaluated by HPLC. For [^55^Co]Co-DOTA-PSMA-617, the radiolabeling also showed a high radiochemical yield and purity above 99.6% (n = 1) and apparent molar radioactivity of 18 MBq/nmol at EOS. Cobalt-57 was used as a surrogate for cobalt-55/58m for the in vitro evaluation and in vivo biodistribution studies.

**Figure 1:**
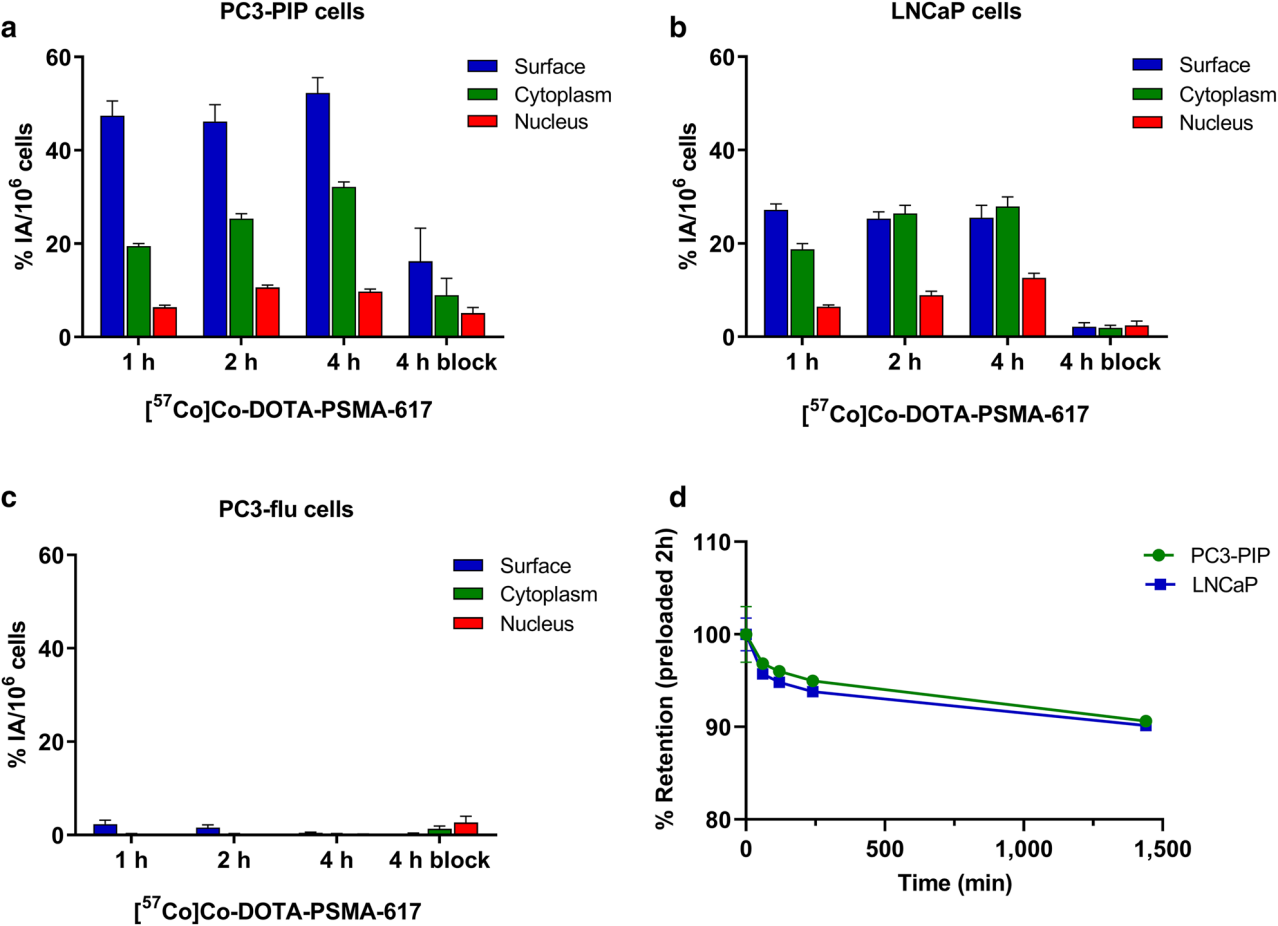
Subcellular distributions of [^57^Co]Co-DOTA-PSMA-617 in (**a**) PSMA-positive PC3-PIP, (**b**) LNCaP, and (**c**) PSMA-negative PC3-flu prostate cancer cells. The subcellular distribution was analyzed at 1, 2, and 4 h, and specificity was performed at 4 h with excess PMPA (4 h block). % IA/10^6^ cells refer to the percentage of injected activity, and the results are normalized to 1 × 10^6^ cells. (**d**) Retention of [^57^Co]Co-DOTA-PSMA-617 for PC3-PIP and LNCaP cell lines over time. Data are presented as the average value (n = 3) ± SEM.

The cellular uptake of [^57^Co]Co-DOTA-PSMA-617 was higher for PC3-PIP than LNCaP cells, while the PSMA-negative PC3-flu cells showed no detectable uptake (Fig. 1). Moreover, the uptake in PC3-PIP and LNCaP cells was PSMA-specific since it could be blocked with an excess of 2-(phosphonomethyl)-pentanedioic acid (PMPA). The highest amount of activity was seen on the cell surface, followed by the cytoplasm and the nucleus, see Fig. 1. The uptake in the cytoplasm at 4 h was 32.1 ± 1.0% IA/10^6^ for PC3-PIP cells, 27.9 ± 2.1% IA/10^6^ for LNCaP cells, and 0.2 ± 0.09% IA/10^6^ for PC3-flu cells. The highest uptake in the nucleus was observed at 4 h and was 9.7 ± 0.6% IA/10^6^, 12.6 ± 1.0% IA/10^6^, and 0.14 ± 0.1% IA/10^6^ for PC3-PIP, LNCaP, and PC3-flu cells, respectively. High retention of the internalized activity in PC3-PIP and LNCaP cells was found, resulting in 90.6 ± 0.4% and 90.1 ± 0.4% retained activity in the cells after 24 h (Fig. 1d).

**Figure 2:**
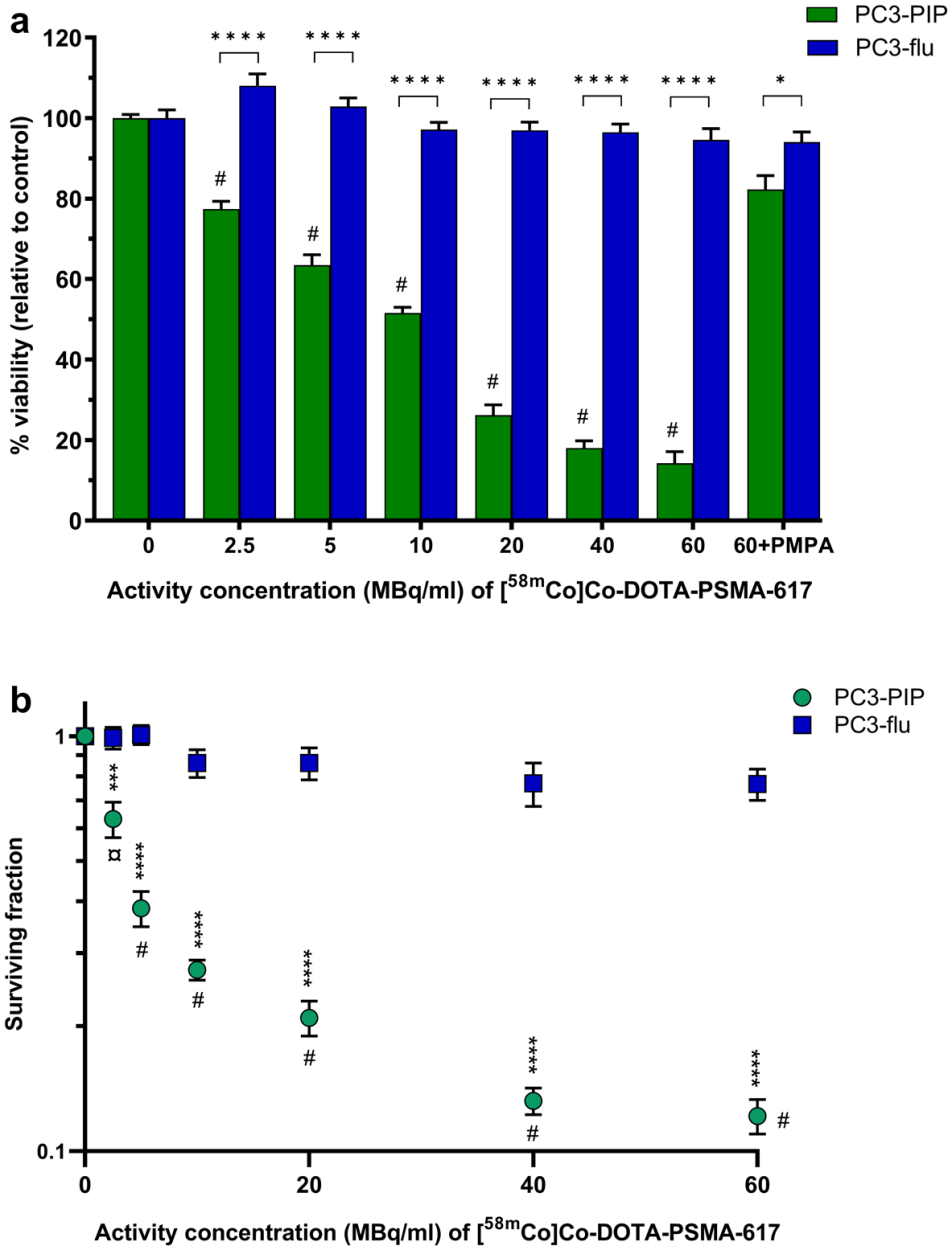
(**a**) Viability of PC3-PIP and PC3-flu cells for increasing activity concentrations of [^58m^Co]Co-DOTA-PSMA-617, showing significant dose-dependent therapeutic effects in PC3-PIP cells (^#^p < 0.0001). Blocking was done with addition of 10 µM PMPA. For each activity concentration, the viability of PC3-PIP cells was significantly lower than that of PC3-flu cells (*p < 0.05; ****p < 0.0001). (**b**) Clonogenic survival of PC3-PIP and PC3-flu cells for increasing activity concentrations of [^58m^Co]Co-DOTA-PSMA-617, showing a significant dose-dependent reduction in survival of PC3-PIP cells compared to untreated control cells (^¤^p < 0.001, ^#^p < 0.0001). No significant reduction in survival was found for PC3-flu cells ***: p<0.001; ****: p<0.0001.

There was a significant dose-dependent decrease in the viability of PC3-PIP cells for all activity concentrations of [^58m^Co]Co-DOTA-PSMA-617 (p < 0.0001). The viability of PC3-PIP cells was significantly lower compared to that of PC3-flu cells (p < 0.0001) for all activity concentrations, and there was no significant decrease in the viability of PC3-flu cells (Fig. 2a). These results were further confirmed by clonogenic assay with [^58m^Co]Co-DOTA-PSMA-617, which showed a significant dose-dependent reduction in the surviving fraction of PC3-PIP cells (2.5 MBq/ml: p < 0.001; 5–60 MBq/ml: p < 0.0001). There was a surviving fraction of 0.12 ± 0.01 for PC3-PIP cells at 60 MBq/ml [^58m^Co]Co-DOTA-PSMA-617, compared to the minor effect in PC3-flu cells (0.77 ± 0.07) at the same activity concentration (p < 0.0001). No significant reduction in survival was observed for PC3-flu cells (Fig. 2b).

### In vivo studies

#### Biodistribution

**Figure 3:**
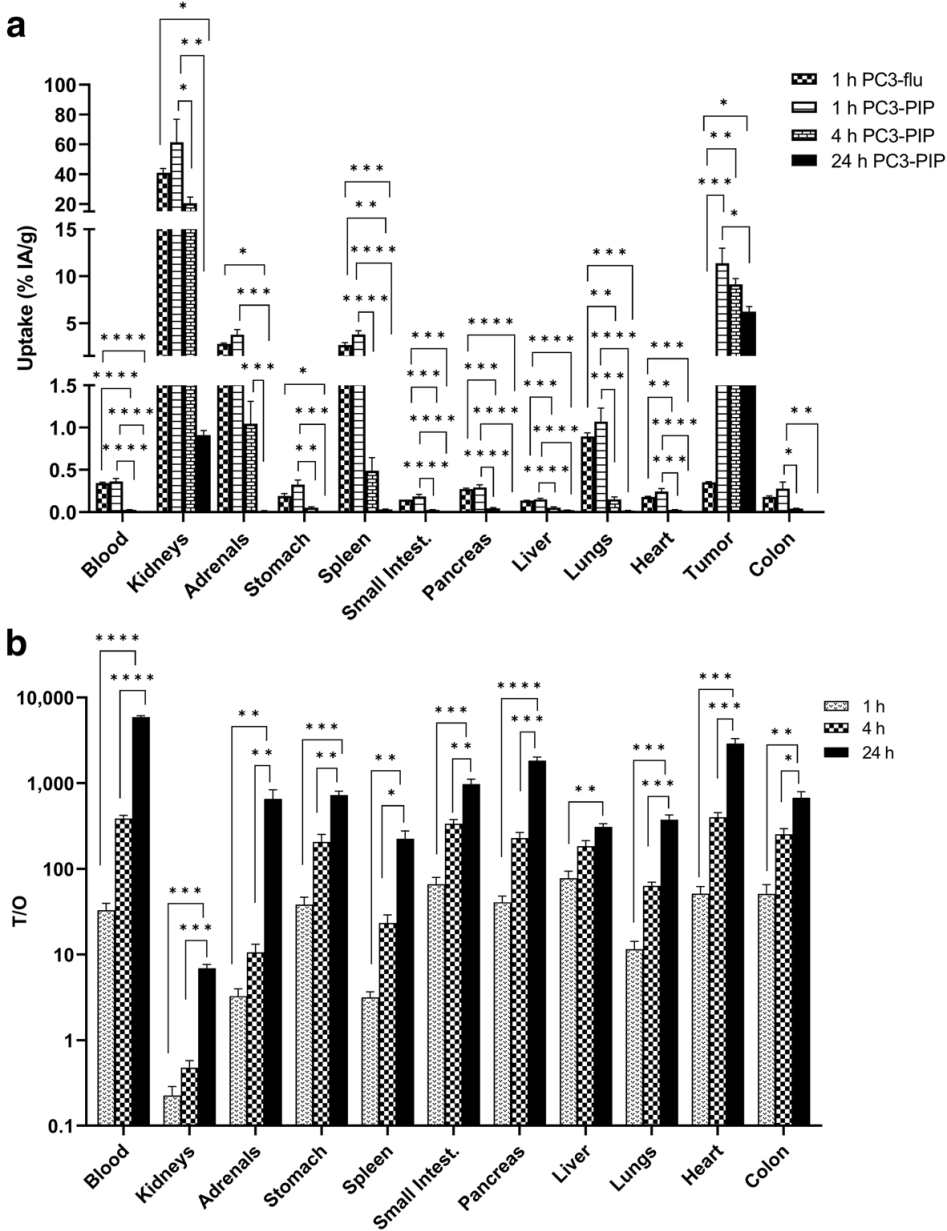
(**a**) Biodistribution of [^57^Co]Co-PSMA-617 in PC3-PIP or PC3-flu tumor-bearing mice (PC3-flu was only analyzed at 1 h pi). There was significantly higher specific tumor uptake in PC3-PIP tumors compared to PC3-flu at 1 h (p < 0.001), and the initial high uptake in kidneys was strongly and significantly reduced over time. (**b**) Tumor-to-organ ratios at 1, 4, and 24 h pi calculated from the biodistribution data showed increasing tumor-to-organ ratios over time. *p < 0.05; **p < 0.005; ***p < 0.0005; ****p < 0.0001.

The ex vivo biodistribution of [^57^Co]Co-DOTA-PSMA-617 was evaluated in tumor-bearing mice (body weight 23.5 g ± 2.2 g, and tumor size 227.1 ± 150 mm^3^ (mean ± SD)) with PC3-PIP cells (1, 4, and 24 h post-injections (pi)) and PC3-flu cells (1 h pi). There was high PSMA-specific tumor uptake at all three time-points for PC3-PIP tumors, 11.4 ± 1.6% IA/g at 1 h pi, 9.4 ± 0.6% IA/g at 4 h pi, and 6.2 ± 0.5% IA/g at 24 h pi (Fig. 3a). The tumor uptake decreased over time, but only significantly between 1 and 24 h (p = 0.04). The tumor uptake in PC3-flu tumors was 0.4 ± 0.007% IA/g 1 h pi, and thus significantly lower than in PC3-PIP tumors (p < 0.001). The kidneys showed the highest uptake at all time-points due to renal clearance of the radiopharmaceutical. It was most pronounced at 1 h pi with 61.4 ± 15.3% IA/g, decreasing over time to 20.5 ± 4.1% IA/g at 4 h pi and significantly from 4 to 24 h to 0.9 ± 0.05% IA/g (p = 0.03). The adrenal glands and spleen also showed uptake after 1 and 4 h pi but cleared significantly at 24 h (Fig. 3a). The overall low uptake in normal tissue resulted in high tumor-to-organ ratios for all organs, which increased over time but only significantly between 1 and 24 h and between 4 and 24 h except for the liver (Fig. 3b). Kidneys displayed the lowest tumor-to-organ ratio, but due to the clearance of [^57^Co]Co-DOTA-PSMA-617, the tumor-to-kidney ratio increased from 0.2 ± 0.06 at 1 h pi to 7.0 ± 0.7 at 24 h (p < 0.001).

#### PET/CT imaging with [^55^Co]Co-DOTA-PSMA-617

**Figure 4:**
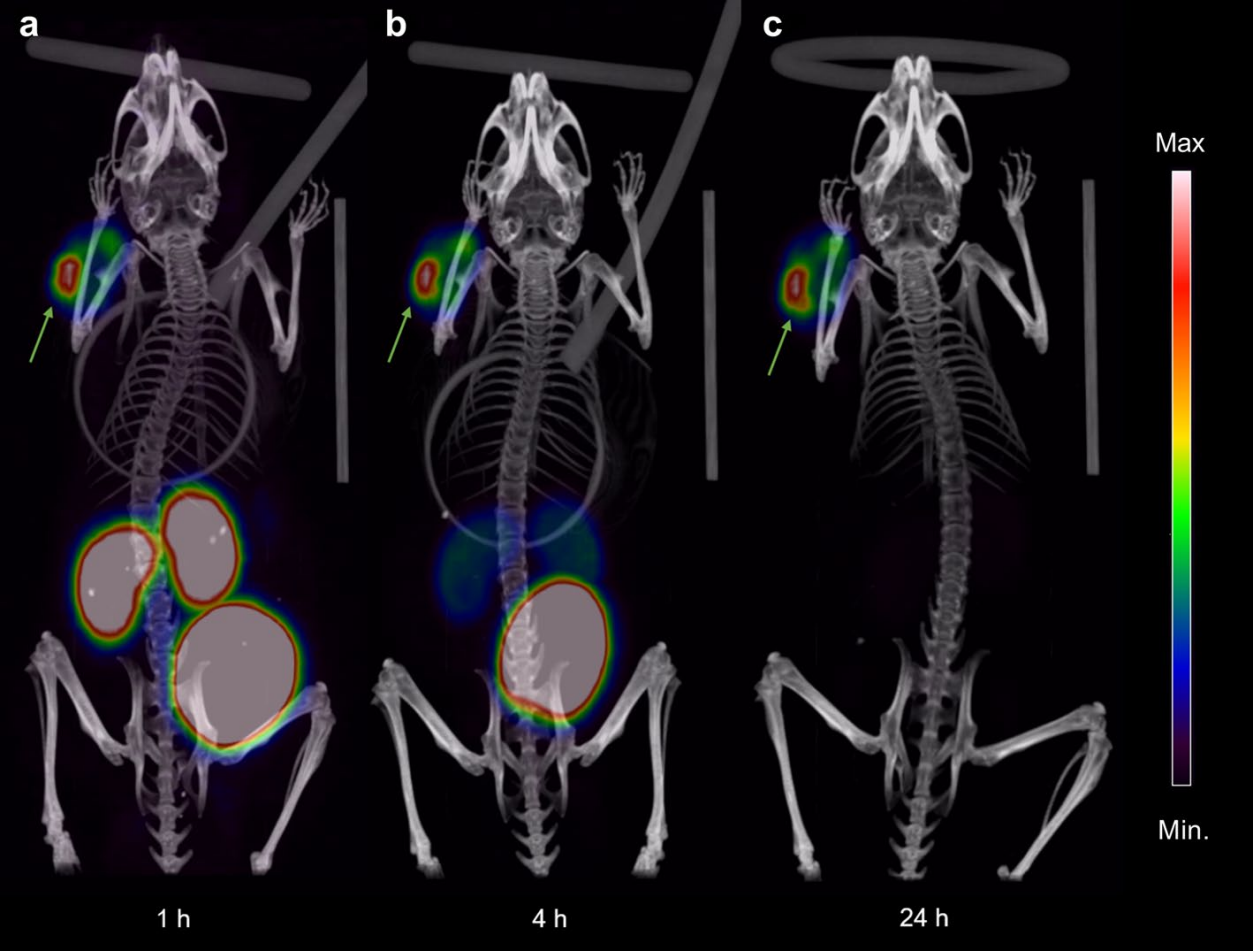
Coronal maximum intensity projection PET/CT images showing uptake and distribution of [^55^Co]Co-DOTA-PSMA-617 in PC3-PIP tumor-bearing NOD-SCID mice at (**a**) 1 h, (**b**) 4 h, and (**c**) 24 h pi. The intensity of the PET data is displayed from zero to maximum tumor uptake. Arrows indicate the subcutaneous PC3-PIP tumor.

The PET/CT imaging of mice with PC3-PIP tumors showed clear visualization of the tumor at all time-points (1, 4, and 24 h) after injection of [^55^Co]Co-DOTA-PSMA-617 (Fig. 4). However, a high accumulation of the activity was seen in the kidneys and bladder at the scans performed after 1 h, which decreased and was less pronounced at the 4 h time-point. Furthermore, the PET images obtained at 24 h pi revealed a very high image contrast between the tumor and background due to no perceptible uptake in normal organs, which confirmed the biodistribution results.

#### In vivo therapy with [^58m^Co]Co-DOTA-PSMA-617

**Figure 5:**
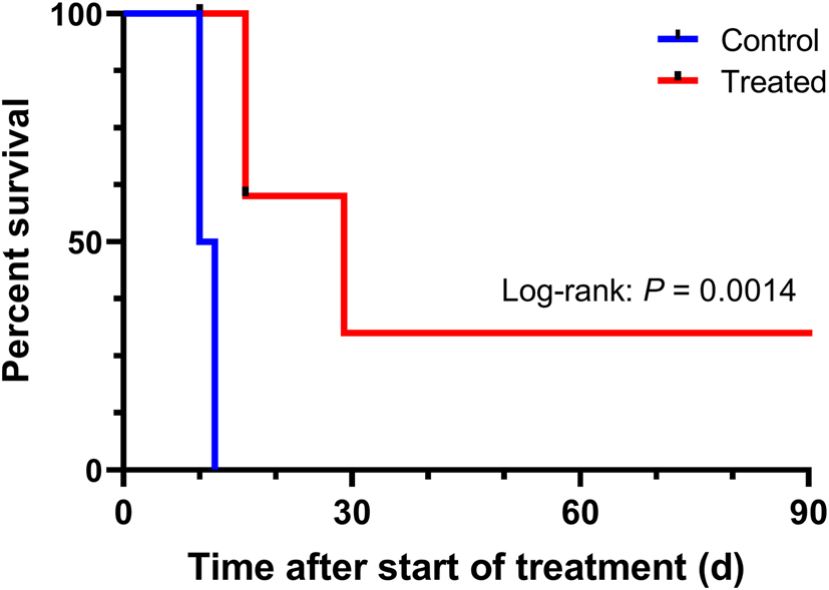
Kaplan–Meier curves for the therapy study showing the percent survival between the treated (n = 4) and control mice (n = 6) with significantly longer median survival for treated mice (p = 0.0014) than for control mice. In one mouse, a complete response was observed.

Twelve mice (control: n = 6, treated: n = 6) with subcutaneous PC3-PIP tumors had equal tumor size and body weight; 40.5 mm^3^ [24.0–56.3] and 24.3 g [23.5–26.5] for treated mice vs. 40.5 mm^3^ [32.0–40.5] and 25.4 g [22.5–26.5] for control mice (p = 0.58 and p = 0.51) on the day of treatment initiation. The results of the therapy study are shown as Kaplan–Meier curves in Fig. 5. Two mice from the treated group had to be censored (days 10 and 16) due to hostility between cage rivals, which resulted in terminal lesions. The control group reached endpoints for tumor size on days 10 and 11. The treated mice had a significantly improved median survival time of 23 days compared to untreated mice, with a median survival time of 11 days (p = 0.0014). One of the four remaining treated mice had a complete response (the tumor disappeared) and was euthanized at the end of the study. Hence, a 110% increase in median survival was obtained.

#### Adverse effects

**Figure 6:**
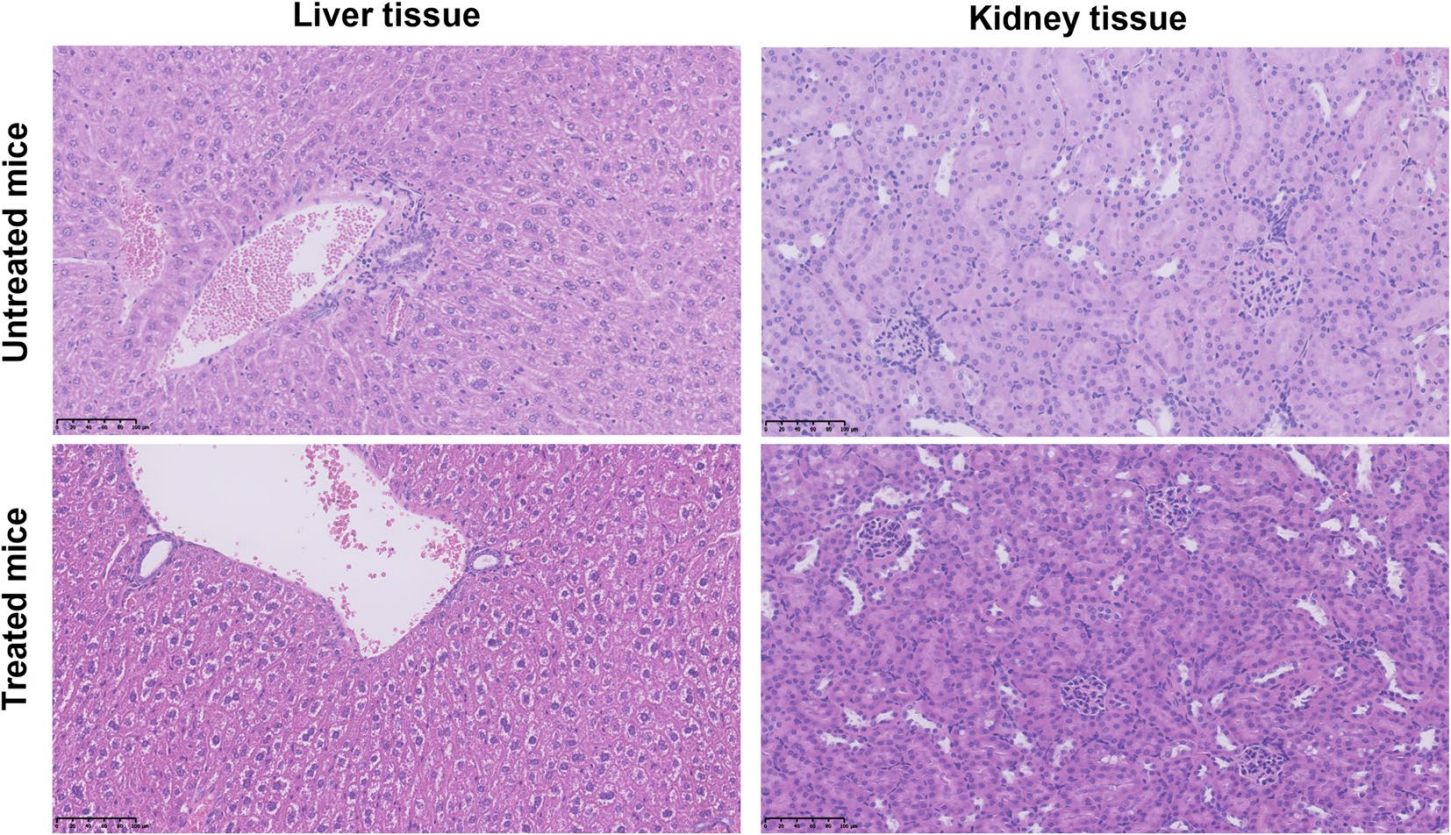
Examples of histopathological images of liver tissue with portal areas (left column) and kidney (renal cortex) tissue (right column) in the treatment study from untreated mice (upper row) and treated mice (lower row), respectively. No signs of tissue damage or difference between the groups were observed. All images are shown in 20 × magnification.

The histopathological assessment of liver and kidney tissue in the treatment study showed no signs of treatment related adverse effects (inflammation, fibrosis, or necrosis). Furthermore, no visible differences were observed between treated and untreated mice (Fig. 6). Hence, the treatment was well tolerated.

## Discussion

Here, the theranostic pair [^55/58m^Co]Co-DOTA-PSMA-617 was investigated for imaging and therapy of prostate cancer in vitro and in vivo. We found a high, specific internalization of [^57^Co]Co-DOTA-PSMA-617 in PSMA-positive cells with 9.7 ± 0.6% IA/10^6^ and 12.6 ± 9.6% IA/10^6^ located in the nucleus after 4 h incubation and a high (> 90%) cellular retention after 24 h for PC3-PIP and LNCaP cells, respectively. Nuclear localization is desirable for the AE to induce lethal double-strand DNA breaks^8,9^. We observed a dose-dependent therapeutic effect in PC3-PIP cells compared to PC3-flu cells in viability and clonogenic assays demonstrating the intended impact of lethal double-strand breaks in the DNA. Auger emitters do not provide cross-fire irradiation like β-particle emitters, which is beneficial since non-targeted tissue is spared from unwanted radiation exposure (e.g. radiation-sensitive bone marrow). However, it might also be a limitation in larger tumors where heterogeneity could yield malignant cells without target receptors, and the AE could be less effective^8^. Our in vitro results are comparable to the findings by Kiess et al. investigating the effect of the PSMA targeting Auger emitting ligand ^125^I-DCIBzL, who also found a high, specific uptake and significant dose-dependent decreased survival^13^.

The ex vivo biodistribution and PET-imaging with [^55^Co]Co-DOTA-PSMA-617 confirmed the in vitro results with high, specific tumor uptake in PC3-PIP tumors compared to PC3-flu tumors. Furthermore, we observed fast excretion of the radioligand over time in normal tissue and a minor and slower decrease in tumor uptake resulting in increasing tumor-to-organ ratios over time.

We and others have previously investigated the use of cobalt-55 for PET-imaging of somatostatin receptors^11,20^, gastrin-releasing peptide receptors^18,21,23^, folate receptors^24^, neurotensin receptors^22,25^, and PSMA^17^, which all found an improved image contrast at late time-point imaging. Moreover, cobalt-55 production is feasible with modern cyclotrons in high yields and purity^15–17^. With this in mind, [^55^Co]Co-DOTA-PSMA-617 is the optimal radioligand for PET imaging in theranostic combination with [^58m^Co]Co-DOTA-PSMA-617.

In the in vivo therapy study, the mice received two treatments (days 7 and 14) with 144 ± 9 MBq (2.3 ± 0.1 nmol) of [^58m^Co]Co-DOTA-PSMA-617 and showed significantly prolonged survival compared to control mice. Other groups also investigated the treatment effect of PSMA-targeting Auger electron-emitting radiopharmaceuticals^12,13^. Kiess et al. evaluated the therapeutic effect of a single treatment with ^125^I-DCIBzL (111 MBq) in vivo. The treatment was given on day 7 after subcutaneous tumor inoculation when the tumor was approximately 3–4 mm. They showed significantly delayed tumor growth compared to the control groups^13^. In another recent study from the same group, Shen investigated the therapeutic effect of a single treatment with ^125^I-DCIBzL (dose levels 0.37–111 MBq)^12^. The treatment was given 7–10 days after iv injection of PC3-PIP tumor cells, where the mice had developed metastases in the liver, kidneys, and bone. The study showed a significant survival benefit in mice receiving doses > 18.5 MBq (10–11 weeks) compared to untreated mice or those receiving a lower dose (< 3.7 MBq) (6 weeks) and no long-term toxicities^12^. Our therapy study’s median survival was remarkably shorter compared to Kiess et al*.* and Shen et al*.* One plausible explanation could be different endpoints in the studies. We euthanized the mice if the tumor reached 12 mm (width or length) or a total tumor volume of 450 mm^3^, whereas Kiess et al. had an endpoint for the tumor size at approx. 15–20 mm^13^. Shen et al*.* euthanized the mice in case of a > 20% drop in body weight or signs of discomfort^12^.

Comparing the dosimetric properties of iodine-125 and cobalt-58m on the cellular scale, the two radionuclides have nearly identical cellular S(N ← N)-values, 2.06e-3 Gy/(Bq s) for cobalt-58m and 2.13e-3 Gy/(Bq s) for iodine-125 with cell nucleus radius of 6 µm^16^. However, the theoretically calculated tumor-to-normal-tissue dose ratios per decay (TND) favor cobalt-58m^16,26^. The TND values for cobalt-58m are almost three times higher than for iodine-125^26^. The high TND suggests a clear benefit for cobalt-58m providing a lower dose to non-targeted tissue and a high absorbed dose to the tumor. On the other hand, the much longer half-life of iodine-125 (59.4 days) compared to 9.04 h for cobalt-58m, could lead to a higher number of cumulated decays in the tumor tissue and thus increase the therapeutic effect of iodine-125. This could explain the increased efficacy observed by Kiess et al*.* However, using iodine-125 in a clinical setting might not be feasible due to the long half-life and associated risk of contamination and generation of high-activity long-lived waste. Hence, the clinical value of [^125^I]I-DCIBzL is questionable.

In PSMA-targeted therapy, the dose-limiting organs are the salivary glands, red bone marrow, and potentially kidneys^27^. Hematologic toxicity is the most common adverse event and a dose-limiting factor, especially in patients with multiple bone metastases^28^. By [^177^Lu]Lu-PSMA therapy, the maximum tissue penetration range is < 1.7 mm, and cross-fire irradiation accounts for most of the unwanted effects in healthy tissue^29,30^. On the contrary, the very short range of AE's emitted by cobalt-58m results in very limited cross-fire into healthy tissue (e.g. red bone marrow from circulating radioactivity), and thus, should enable treatments with high activity levels to increase the therapeutic effect. In the current study, bone marrow toxicity was not assessed, but no weight loss or general discomfort was observed in the treated mice. The study by Kiess et al. included a complete blood count four days post-treatment with ^125^I-DCIBzL and only found a mild acute inflammation response^13^. Similar results were found by Shen et al., who evaluated the bone marrow toxicity by blood counts and histopathology and found no long-term toxicity^12^. These results indicate that the bone marrow toxicity is limited within the radioactivity levels administered in this study. In patients, PSMA-targeted therapy often causes severe xerostomia due to high unspecific accumulation of the PSMA-radioligand in salivary glands, with the mechanism of the latter remaining unclear^31^. In the present study, PET images did not show any uptake of [^55^Co]Co-DOTA-PSMA-617 in the salivary glands of the mice, which agrees with previously published studies and is primarily due to differences between species^32^. Furthermore, no adverse effects was reported by Shen et al. who included necropsy with histopathology of the salivary glands^12^. However, rodent models might not be appropriate to evaluate toxicity to salivary glands due to the decreased PSMA expression compared to humans^32^. In the present study, we evaluated potential side effects by histopathology (Fig. 6). We did not observe any differences between treated and untreated mice in the therapy study. This could indicate that the maximum tolerated dose was not reached and aligns with the theoretical knowledge of beneficial radiobiological properties for Auger emitters when delivered and internalized to cancer cells^8–10^. PSMA-targeted AE therapy can not be directly compared to lutetium-177 therapy due to the different dosimetric properties. The clinical perspective for [^58m^Co]Co-DOTA-PSMA-617 targeted therapy will be most beneficial for patients with small tumors or in advanced disease with multiple distant metastases due to the highly localized (i.e. subcellular) effect^26^.

From a logistic perspective, the theranostic cobalt-55/58m pair can be produced in high amounts locally using small PET-cyclotrons equipped with solid target systems^14,15^ or potentially distributed from centralized production facilities similar to the distribution of marketed copper-64 chloride (Cuprymina).

Cobalt-58m decays solely to the long-lived ground state cobalt-58g, which has a half-life of 70.9 days^15,16^. Therefore, if no excretion of this radionuclide occurs in vivo, the generated cobalt-58g activity will be approx. 0.5% of the administered cobalt-58m activity when all cobalt-58m has decayed. However, we have demonstrated that the radiocobalt-DOTA complex is stable in phosphate-buffered saline during the cobalt-58m decay, and thus, [^58g^Co]Co-DOTA-PSMA-617 is formed. Furthermore, considering the biodistribution results in Fig. 3 and the PET/CT scans in Fig. 4, more than 98% of the radioligand has been excreted at 24 h pi. Therefore, the remaining [^58g^Co]Co-DOTA-PSMA-617, primarily located in the tumor, will be less than 0.01% of the injected activity and, thus, negligible.

The limitations include the small number of animals in the treatment group of the therapy study because of the censoring of two mice. Despite this, we found a significantly longer survival than the control group. Another limitation of our study was the evaluation of potential adverse effects, which could have included a more exhaustive assessment of possible toxicities. A complete blood analysis at different time points and histopathological evaluation of salivary glands could be included in a future study with larger animal groups.

## Conclusion

The theranostic pair [^55/58m^Co]Co-DOTA-PSMA-617 demonstrated excellent in vitro and in vivo properties and showed significant therapeutic effects in mice xenografted with prostate cancer cells with no toxicities observed. Hence, it should be further investigated for future clinical translation.

## Materials and methods

### Radiolabeling

Cobalt-55 for imaging and cobalt-58m for therapy were prepared in-house as described^11,14,16^. Cobalt-57 (t½=271.3 days) was used as a convenient surrogate for cobalt-55/58m for the in vitro evaluation and in vivo biodistribution studies. Carrier-free cobalt-57 (0.1 M HCl, 370 MBq) was purchased from PerkinElmer (Skovlunde, DK).

DOTA-PSMA-617·TFA (PSMA-617) was purchased from ABX (Radeberg, GE). Deionized water (resistivity = 18.2 MΩ·cm) was used for preparing reagent solutions. Microwave heating was used for radiolabeling using a PETWave (CEM Corporation, US). Sodium acetate buffer (NaOAc) (pH 4.5, 0.4 M) for radiolabeling was prepared from TraceSelect grades anhydrous sodium acetate (≥ 99.999%) and acetic acid (≥ 99.0%) supplied by Merck (Darmstadt, GE) and deionized water. The radiochemical yield and purity were determined by high-performance liquid chromatography (HPLC) and instant TLC^17^. For animal studies, the radioligands were diluted with sterile-filtered phosphate-buffered saline (PBS) containing 0.1% bovine serum albumin (BSA). The radiolabeling with cobalt-55/57 was performed as described previously^17^. For radiolabeling of DOTA-PSMA-617 with cobalt-58m; ^58m^CoCl_2_ (50 µl 0.04 M HCl, 1555 ± 176 MBq) and 85 µl PSMA-617 (0.25 µg/µl) in sodium acetate buffer (0.4 M, pH 4.5) were mixed in a small glass vial, sealed, vortexed and heated for 120 s at 90 °C by dynamic microwave irradiation^11,16^. To evaluate the radio-complex stability over time, the [^58m^Co]Co-DOTA-PSMA-617 complex was kept in 0.1% BSA in PBS immediately after labeling and stored at room temperature. HPLCs were performed at the following time points after labeling: 0 h, 24 h, 48 h, 96 h, and eight days.

### In vitro studies

The isogenic human prostate adenocarcinoma cell lines PC3-PIP (PSMA-positive) and PC3-flu (PSMA-negative) were obtained from Dr. Warren Heston (Cleveland Clinic, Cleveland, Ohio, US). The human prostate adenocarcinoma cell line LNCaP was purchased from CLS Cell Lines Services (Eppelheim, GE). The cells were cultured in complete medium consisting of RPMI 1640 supplemented with 10% fetal bovine serum and 1% penicillin/streptomycin (P/S) (all from ThermoFisher, Roskilde, DK). The cells were maintained at 37 °C in a humidified atmosphere with 5% CO_2_. All in vitro studies were performed in triplicates.

#### Subcellular distribution and efflux

PC3-PIP, PC3-flu (both 1 × 10^5^), and LNCaP (2.5 × 10^5^) cells were seeded in 24-well plates, the latter on poly-lysine-coated plates, 24 h before the experiment. The next day, cells were washed twice with incubation medium (RPMI 1640 with 1% BSA) and incubated with 20 kBq/ml [^57^Co]Co-DOTA-PSMA-617 in incubation medium for 1, 2, and 4 h at 37 °C. The experiment was stopped by removing the incubation medium, and the cells were washed twice with cold PBS. To remove surface-bound radioligand, the cells were incubated on ice for 5 min in cold acid wash (0.2 M NaOAc, 0.5 M NaCl pH 2.5). Next, the cells were fractionated into cytoplasm and nuclei by incubating for 5 min on ice in cold nuclei EZ prep lysis buffer (Merck, Darmstadt, GE). Finally, the cells were scraped and centrifuged 500×*g* for 5 min at 4 °C. The supernatant (cytoplasm) was transferred to new tubes, and the pellet (nuclei) was resuspended in PBS. The radioactivity of the surface-bound, the cytoplasm, and the nuclei was measured in a 2470 Wizard Automatic Gamma Counter (PerkinElmer). Non-specific binding was assessed by co-incubating with 2 µM PMPA.

For efflux studies, PC3-PIP (6 × 10^5^) and LNCaP (1 × 10^6^) cells were seeded in 6-well plates, the latter on poly-lysine-coated plates, and allowed to adhere overnight. The cells were incubated as described above, and the cellular uptake was stopped after 2 h by removing the incubation medium. Unbound radioactivity was removed by washing the cells three times with cold PBS, and surface-bound activity was removed with cold acid wash, as described above. The cells were washed with incubation medium and returned to the incubator in fresh medium. At 1, 2, 4, and 24 h, the medium was collected and replaced with new incubation medium. The experiment was terminated by incubating the cells with 1 M NaOH to extract the remaining cell-associated radioligand. The radioactivity of the surface-bound, the collected medium, and the cells were measured in a 2470 Wizard Automatic Gamma Counter.

#### Cell viability and clonogenic assay

PC3-PIP and PC3-flu cells were incubated with 0–60 MBq/ml of [^58m^Co]Co-DOTA-PSMA-617 for 24 h. The next day, cells were trypsinized and counted; after that, 1000 cells/well were seeded in 96-well plates in 150 µL complete medium. On day seven after seeding, the cell viability was evaluated by adding 20 µl CellTiter-Blue (Promega, Nacka, Sweden). Fluorescence was measured at (Ex520nm/Em580–640nm) in the GloMax Explorer (Promega). The viability was normalized to control cells and displayed as percentages. Clonogenic assay was performed by seeding PC3-PIP and PC3-flu cells (1 × 10^5^) in 24-well plates and allowed to adhere overnight. Increasing activity concentrations (0, 2.5, 5, 10, 20, 40, 60 MBq/ml) of [^58m^Co]Co-DOTA-PSMA-617 was added in incubation medium and incubated for 24 h. The incubation medium was removed, and the cells were trypsinized, counted, and seeded in complete medium for ten days. The colonies were stained with 0.05% Crystal Violet and colonies larger than 50 cells were measured using the colony counter from the ImageQuantTL software (GE Healthcare, Brondby, DK). Surviving fractions were normalized to control plates and displayed as percent survival compared to controls.

### In vivo studies

All animals were housed under specific pathogen-free (SPF) conditions with individually ventilated caging (IVC) in groups of 3–5 in an enriched environment. The housing was done under standardized conditions (22–24 °C temperature; 55% ± 15% humidity) on a 12 h light/12 h dark cycle and fed a standard diet and unrestricted access to water ad libitum.

#### Ethics approval

All animal experiments were performed according to the national legislation on protecting laboratory animals and approved by the Danish Animal Experiment Inspectorate (approval number: 2016-15-0201-01027). Furthermore, the animals were housed and handled according to good animal ethics and in compliance with the 3Rs principles (Replacement, Reduction, and Refinement) and the ARRIVE guidelines 2.0^33,34^.

#### Biodistribution

Fifteen male non-obese diabetic/severe combined immunodeficiency (NOD-SCID) mice (6–8 weeks old) were obtained from an in-house breeding program. Twelve mice were subcutaneously inoculated with 5 × 10^6^ PC3-PIP cells and three with 5 × 10^6^ PC3-flu cells at the left shoulder. Eight days after inoculation, the animals were anesthetized under isoflurane and placed on a heating pad (37 °C), and injected via the tail vein with [^57^Co]Co-DOTA-PSMA-617 (55.0 ± 3.2 kBq, 17.0 ± 1.0 pmol in 100 µl). The mice were euthanized by cervical dislocation under deep isoflurane anesthesia in groups (n = 4) at different time points (1, 4, and 24 h, respectively pi). The group (n = 3) with PC3-flu cells was euthanized at 1 h pi. All tissue of interest was collected, weighed, and the amount of radioactivity was measured using a 2470 Wizard Automatic Gamma Counter. The radioactivity uptake in the organs was calculated as the percentage injected activity per gram of tissue (% IA/g), and tumor-to-organ ratios were calculated.

#### PET/CT imaging with [^55^Co]Co-DOTA-PSMA-617

Small-animal PET/CT imaging was performed on a Siemens INVEON multimodality preclinical scanner in docked mode (Siemens, Knoxville, TN, US). Two male NOD-SCID mice (in-house breeding) were subcutaneously inoculated with 4 × 10^6^ PC3-PIP cells at the left shoulder twelve days before imaging. For the imaging, the mice were anesthetized with a mixture of 1.5–2% isoflurane in 100% oxygen and placed on a heating pad before injection of [^55^Co]Co-DOTA-PSMA-617 (3.4 ± 1.5 MBq, 180 ± 20.0 pmol, 100 µl) in the tail vein. At 1, 4, and 24 h pi, the mice were re-anesthetized and placed feet first in a prone position on a heated PET/CT animal bed. The respiration and temperature were monitored during imaging using the BioVet system (M2M Imaging, Cleveland, Ohio, US). The imaging protocol included a two-bed CT for anatomic orientation and attenuation correction performed with full rotation, 360-degree projections, and exposure of 80 kV and 500 µA. A static PET scan immediately followed the CT scan with a duration of 15 min (1 and 4 h pi) and 30 min (24 h pi). CT and PET images were co-registered using a transformation matrix and reconstructed using an OSEM3D/SP-MAP algorithm (2 OSEM and 18 MAP iterations, matrix 128 × 128) and scatter correction, resulting in a final target resolution of 1.5 mm. PET data were analyzed using the dedicated imaging analysis software: Inveon Research Workplace (Siemens Medical Solutions Malvern, PA, USA). The scans are presented as maximum intensity projections adjusted to display a color scale from 0 to the maximum tumor uptake value in the actual scan.

#### [^58m^Co]Co-DOTA-PSMA-617 therapy

Twelve male BALB/c-nu immunodeficient mice (age 4–6 weeks; Taconic Bioscience, Europe) were subcutaneously inoculated with 1 × 10^6^ PC3-PIP cells at the left shoulder. On day 7, after tumor inoculation, the mice were divided into two groups with equal visual tumor sizes. One group (n = 6) was injected iv with [^58m^Co]Co-DOTA-PSMA-617 (144.0 ± 9.0 MBq, 2.3 ± 0.1 nmol, 150 µl), and the other group (n = 6) with 150 µl saline. One week later, the treatment was repeated (day 14 after inoculation). The mice were monitored by body weight and tumor size measurements (digital caliper) every second day. At the end of the observation period (90 days), or when they reached one of the endpoints (tumor volume > 450 mm^3^, tumor length or width > 12 mm, weight loss > 20%, or tumor ulceration), the mice were euthanized. After euthanization, the kidney and liver were collected for histopathological analysis and fixed in formalin. After fixation, the organs were sliced and paraffin-embedded. Tissue sections were deparaffinized and stained with hematoxylin and eosin (H&E). The Dako CoverStainer (Agilent, Santa Clara, CA, USA) was used for the H&E stainings. Tissues were assessed by two pathologists.

### Statistics

Data were analyzed using GraphPad Prism (version 9.2, GraphPad Software Inc.). Statistic analysis was performed using multiple unpaired *t* tests or two-way ANOVA with Holm-Sidak correction for multiple comparisons to determine significant statistical differences (p < 0.05). All p-values were adjusted for multiple comparisons. Descriptive statistics for in vitro and biodistribution data are presented as mean values using standard error of means (SEM). Descriptive statistics for in vivo data are presented as median and range. Survival was analyzed by Kaplan-Meyer analysis and log-rank (Mantel-Cox) test.

## Data Availability

The datasets generated and analyzed during the current study are available from the corresponding author on reasonable request.

## Abbreviations

PSMA: Prostate-specific membrane antigen
AE: Auger electrons
HPLC: High-performance liquid chromatography
TLC: Thin layer chromatography
NOD-SCID mice: Non-obese diabetic/severe combined immunodeficient mice
% IA/g: Percentage of injected activity per gram of tissue
SEM: Standard error of means
SD: Standard deviation
PMPA: 2-(phosphonomethyl)-pentanedioic acid
TND: Tumor-to-normal-tissue dose ratios per decay
MIP: Maximum intensity projection
